# Integrating Phenotypic Search and Phosphoproteomic Profiling of Active Kinases for Optimization of Drug Mixtures for RCC Treatment

**DOI:** 10.3390/cancers12092697

**Published:** 2020-09-21

**Authors:** Judy R. van Beijnum, Andrea Weiss, Robert H. Berndsen, Tse J. Wong, Louise C. Reckman, Sander R. Piersma, Marloes Zoetemelk, Richard de Haas, Olivier Dormond, Axel Bex, Alexander A. Henneman, Connie R. Jimenez, Arjan W. Griffioen, Patrycja Nowak-Sliwinska

**Affiliations:** 1 Angiogenesis Laboratory, Department of Medical Oncology, Amsterdam UMC, Vrije Universiteit Amsterdam, Medical Oncology, Cancer Center Amsterdam, De Boelelaan 1117, 1081HV, 1182 DB Amsterdam, The Netherlands; judy.vanbeijnum@gmail.com (J.R.v.B.); bobberndsen@hotmail.com (R.H.B.); wong1981@live.nl (T.J.W.); l.reckman@outlook.com (L.C.R.); r.dehaas@amsterdamumc.nl (R.d.H.); aw.griffioen@vumc.nl (A.W.G.); 2 Molecular Pharmacology Group, School of Pharmaceutical Sciences, University of Geneva, 1211 Geneva, Switzerland; andrea.weiss07@gmail.com (A.W.); Marloes.Zoetemelk@unige.ch (M.Z.); 3 Institute of Pharmaceutical Sciences of Western Switzerland, University of Geneva, 1211 Geneva, Switzerland; 4 Department of Medical Oncology, Amsterdam UMC, Vrije Universiteit Amsterdam, Cancer Center Amsterdam, De Boelelaan 1117, 1081HV, 1182 DB Amsterdam, The Netherlands; s.piersma@amsterdamumc.nl (S.R.P.); a.henneman@amsterdamumc.nl (A.A.H.); c.jimenez@amsterdamumc.nl (C.R.J.); 5 OncoProteomics Laboratory, Amsterdam UMC, Vrije Universiteit Amsterdam, Cancer Center Amsterdam, 1081 HV Amsterdam, The Netherlands; 6 Department of Visceral surgery, Lausanne University Hospital and University of Lausanne, 1015 Lausanne, Switzerland; olivier.dormond@chuv.ch; 7 Royal Free London NHS Foundation Trust, Renal Cancer Centre, University College London, Division of Surgical and Interventional Science, London NW3 2QG, UK; a.bex@nki.nl; 8 Netherlands Cancer Institute, 1066 CX Amsterdam, The Netherlands; 9 Translational Research Centre in Oncohaematology, 1211 Geneva, Switzerland

**Keywords:** combination treatment, drug-drug interactions, drug-target interaction, synergy, carcinoma, RCC

## Abstract

**Simple Summary:**

Renal cell carcinoma (RCC) cancer is among the ten most common malignancies, and frequently presents as metastatic disease (mRCC). For these patients, systemic treatment is in order, but mRCC is often highly heterogeneous, and resistant to conventional therapies, or acquires resistance over time. Application of a combination of targeted therapeutic agents can tackle these problems, however, experimental optimization is not feasible given the enormous number of possible drug- and dose-combinations. We used a phenotypic approach, the streamlined-feedback system control (s-FSC) technique, which does not use a priori information on the mechanism of action of drugs. Using a number of searches, this method selects for optimized drug combinations (ODC) given at low doses (ED_5-25_), that can act synergistically. This way, we selected effective ODC for different RCC cell lines. Analysis of kinase activity was performed to provide mechanistic insight into the ODC action, and to further improve the found drug combinations.

**Abstract:**

Combined application of multiple therapeutic agents presents the possibility of enhanced efficacy and reduced development of resistance. Definition of the most appropriate combination for any given disease phenotype is challenged by the vast number of theoretically possible combinations of drugs and doses, making extensive empirical testing a virtually impossible task. We have used the streamlined-feedback system control (s-FSC) technique, a phenotypic approach, which converges to optimized drug combinations (ODC) within a few experimental steps. Phosphoproteomics analysis coupled to kinase activity analysis using the novel INKA (integrative inferred kinase activity) pipeline was performed to evaluate ODC mechanisms in a panel of renal cell carcinoma (RCC) cell lines. We identified different ODC with up to 95% effectivity for each RCC cell line, with low doses (ED_5–25_) of individual drugs. Global phosphoproteomics analysis demonstrated inhibition of relevant kinases, and targeting remaining active kinases with additional compounds improved efficacy. In addition, we identified a common RCC ODC, based on kinase activity data, to be effective in all RCC cell lines under study. Combining s-FSC with a phosphoproteomic profiling approach provides valuable insight in targetable kinase activity and allows for the identification of superior drug combinations for the treatment of RCC.

## 1. Introduction

Renal cell carcinoma (RCC) is one of the ten most common malignancies [1]. Patients who present early or localized stage RCC have a high five-year survival (over 90%), whereas for patients with metastatic RCC this rate drops dramatically [2]. Standard therapy for localized RCC is surgery, however many patients develop metastasis making the disease management difficult [3]. Due to its heterogeneity, RCC is intrinsically resistant to chemotherapy and radiotherapy [4]. Mutations in different genes, e.g., *VHL*, *p53*, *PTEN*, and *mTor*, have been associated with the development of RCC [5]. Furthermore, during the course of tumor progression subclones may arise, carrying a different set of mutations [5]. In addition, selection of resistant subclones can occur during the course of therapy, either as a consequence of additional acquired mutations, or due to altered protein expression or metabolic changes [5,6,7,8]. As such, there is need for therapy regimens tailored to these divergent phenotypes. 

Since their introduction, targeted therapies have caused a renaissance in the therapy of RCC [9]. Tyrosine kinase inhibitors (sunitinib, sorafenib, pazopanib and axitinib) [10], the humanized anti-VEGF antibody bevacizumab (with interferon-α) [11], and two mammalian target of rapamycin (mTOR) complex 1 kinase inhibitors (temsirolimus and everolimus) are currently available for treatment. All these drugs mainly target only two signaling pathways with more or less specificity, i.e,. the VEGF- and mTOR signaling pathways. These pathways regulate key events in cellular survival, proliferation, metabolism and angiogenesis, critical processes in metastatic RCC (mRCC). Several years of effort testing these compounds as monotherapies resulted in the insight that the prolongation of progression free- and overall-survival has reached a plateau. Recently, cabozantinib, an inhibitor of VEGF-R, MET, and AXL, demonstrated progression free- and overall-survival advantages over sunitinib, and received FDA approval for first-line mRCC treatment [12]. However, the still relatively limited activity of single drug therapies is mainly due to toxicity [13], acquired drug resistance [14] and possibly even by enhancement of metastasis [15]. To date, many preclinical and clinical efforts have focused on combining existing agents or sequencing them to maximize their impact on clinical outcomes. Large studies such as the RECORD [16], INTORACT [17], TORAVA [18] or KEYNOTE-426 [19] investigated combination strategies but rather showed their lack of benefit [20]. Novel strategies for finding optimal drug combinations are therefore highly needed.

Different challenges arise when optimizing multi-drug combinations: (i) the innately complex nature of a biological system, which makes it virtually impossible to predict optimal drug combinations based on single drug-target interaction information alone, (ii) the large number of possible drug combinations that exist when multiple drugs are considered at multiple concentrations, and (iii) the uncertain in vivo toxicity profile of combination therapies. We demonstrate that these challenges can be addressed through the implementation of the validated streamlined-feedback system control (s-FSC) technique, which provides a systematic and quantitative approach of determining optimized drug combinations (ODC) and their doses to obtain a desired therapeutic outcome. The s-FSC is a phenotypic approach, and therefore does not require any prior mechanistic information, in order to rapidly converge upon ODC. In contrast to other approaches based on pharmacogenetics or high-throughput screening, the s-FSC is based on the statistical design of experiment (DoE) together with regression analysis. In only three experimental steps s-FSC identifies synergistic and/or additive drug combinations and optimized doses of the selected compounds [21,22]. As we have previously shown, this allows us to define the best tailored combination of a selection of drugs for a given cell type under study, without the need to have intricate knowledge of their mechanism of action.

The search for drug combinations through applied statistical methods has evolved in recent years. Tan et al. first devised an F-test to evaluate joint actions of two compounds [23], which was later extended with interaction surfaces for evaluation of additive and synergistic effects [24,25]. In this study, we performed the s-FSC-based screen in five genetically different RCC cell lines in order to mimic a patient tailored approach and to identify the cell line-specific ODC, as well as a “common” drug mixture potent in all tested RCC cell lines. In parallel, we performed a phosphoproteomics profiling approach to unravel signaling events in the cells, in order to predict optimal drugs by the profile of active kinases [26]. We demonstrate that drug combinations signal through different pathways than is known for the mono-targeted therapy and that the combination of s-FSC with a phosphoproteomic profiling approach appears to identify superior drug combinations for the treatment of RCC.

## 2. Results

### 2.1. Phenotypic Search Identifies Cell Type Specific Optimal Three-Drug Combinations 

**Figure 1:**
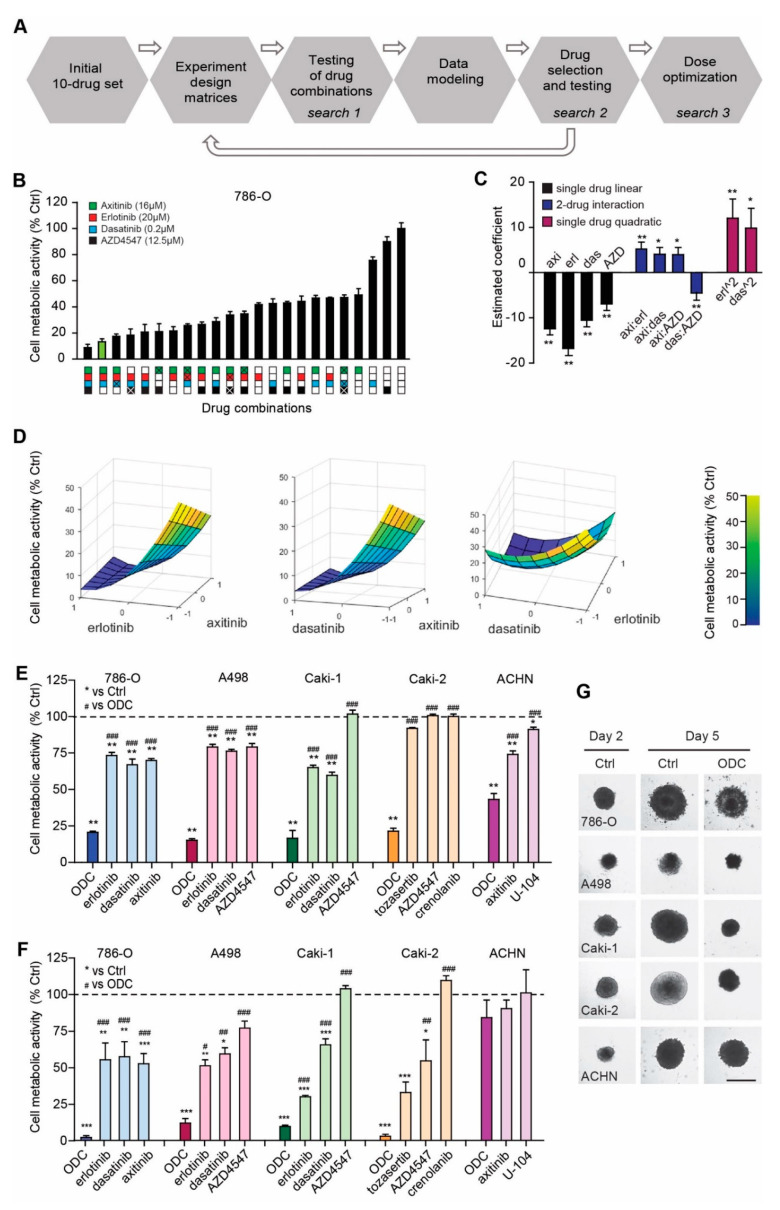
Selection of optimal drug combinations (ODC) using streamlined-feedback system control (s-FSC). (**A**) Schematic of s-FSC. For a selection of 10 drugs, dose–response curves are generated in the cell types of interest. Mathematic modeling and regression analysis of experimental data drive towards an optimal drug combination of 3 to 4 drugs in 3 to 4 sequential rounds of experimentation. (**B**) Experimentally tested combinations from searches 3 and 4, are shown in order of effectiveness. Colored squares below the Y-axis denote drug combinations, with complete fills representing the indicated (IC_20_) concentrations and fills with “X” represent half of the drug concentration. White squares indicate absence of the drug. The best performing 3-drug combination is indicated in green and designated ODC for further reference. Experiments (N = 2) were performed in triplicate and mean ± SEM are shown. (**C**) Regression coefficients describing single linear drug effects, two-drug interaction terms and single drug quadratic effects based on the data obtained under (B). Negative single drug regression coefficients indicate that increasing the dose of that drug increases the inhibition of cell viability. Negative two-drug interaction values denote synergy. Quadratic single drug interactions are a measure of sensitivity to dose changes. (**D**) Response surfaces of drugs in the final ODC for 786-O. 2-drug responses are shown, with on the x-axis 1 = highest concentration, 0 = lower concentration, −1 = no drug added, and the remaining cell metabolic activity on the y-axis color coded. (**E**,**F**) ODC and monotherapy for all renal cell carcinoma (RCC) cell lines tested in this study, in 2D monolayers (**E**) and heterotypic 3D spheroids (**F**). Experiments (N = 2–5) were performed in triplicate and mean ± SEM are shown. (**G**) Representative images of spheroids before and after treatment. Scale bar = 600 µm. #: *p* < 0.05, ##: *p* < 0.025, ###: *p* < 0.01, *: *p* < 0.05, **: *p* < 0.025, ***: *p* < 0.01.

A phenotypically-driven search was performed in order to identify cell type specific optimized drug combinations (ODC) using the previously validated streamlined-FSC (s-FSC) methodology [21,22]. Briefly, the s-FSC approach is based on the use of the design of experiment (DoE) approach, and response surface modelling (Supplementary Methods) that allows for the rapid identification of ODC containing multiple (two to four) drugs (Figure 1A). A set of ten clinically used and experimental drugs (axitinib, erlotinib, dasatinib, RAPTA-C, AZD-4547, BEZ-235, volasertib, tozasertib, U-104, crenolanib) was selected for this screen (Figure S1.1.1, Table S1.1), to target a broad array of pathways. Drug doses used in the optimization procedure were selected based on cell line specific dose response curves (Figure S1.1.2) and a series of orthogonal array composite design (OACD) matrices were used to select drug combinations to be screened in simple in vitro assays of cell metabolic activity (cell viability) inhibition. In particular, three sequential search rounds were performed exploring mixtures of 10, 7 or 4 drugs, respectively, applied at maximum doses of IC_20_ (the dose where 20% of maximal inhibition is observed). This platform was applied independently to each of the six cell lines investigated in this study, including three clear cell RCC (ccRCC) cell lines, i.e., 786-O, A498, Caki-1 and two papillary RCC (pRCC) cell lines, i.e., Caki-2, ACHN [27]. Since RCC is highly vascularized, the s-FSC search was performed also in human endothelial cell line, EC-RF24 [28] (Table S1.2, Figure S1.2.1–6).

Analysis of experimental data was performed using regression analysis and response surface modeling (Figure 1B–D). Figure 1B displays the effects of the different combinations of 3 and 4-drug combinations, tested in 2 concentrations, in ascending order. The final ODC, depicted in green, was chosen based on the lowest drug doses that showed maximum synergy. The compounds showing strong single drug activity and/or synergistic interactions were identified based on estimations of each drug’s main effects and drug interaction terms. More specifically, strong single drug activities, denoted by a large negative single-drug coefficient, and synergistic drug interactions, denoted by negative interaction terms, are selected for, while antagonistic drug interactions, denoted by positive drug interaction terms, are selected against. In all ccRCC lines, the high efficacy of the ODC was primarily driven by high single drug activities, predominantly of erlotinib, dasatinib and AZD4547 (negative coefficients of single drug linear effects, Figure 1C, Figure S1.2.1–3), which constituted the final ODC of both A498 and Caki-1. We based our decision to continue in Caki-1 with the best combination without RAPTA-C due to its hormetic behavior [29], i.e., RAPTA-C, alone and in combination with AZD4547 actually stimulated the growth of Caki-1 cells (Figure S1.2.3). In 786-O cells, axitinib was a stronger component in the ODC as compared to AZD4547, despite antagonistic interactions with the other drugs. Caki-2-specific ODC contained, tozasertib, AZD4547 and crenolanib with strong single drug activities and synergistic interactions between tozasertib and crenolanib, as well as between AZD4547 and crenolanib. Remarkably, the ACHN-specific ODC contained only two drugs with synergistic interaction, i.e., axitinib and U-104 (Figure 1E). The majority of drug interactions in this cell line were antagonistic, limiting the selection of effective higher-order drug combinations (Figure S1.2.1–5). Finally, the s-FSC-based optimization in endothelial EC-RF24 cells identified the ODC consisting of BEZ-235, dasatinib and AZD4547, with significant synergistic interaction between dasatinib and AZD4547 (Figure S1.2.6). 

In order to confirm the activity of the ODC optimized in 2-dimentional (2D) cell cultures (Figure 1E), we prepared heterotypic 3D co-cultures consisting of RCC cells and human dermal fibroblasts (HDFA; ratio 3:1), supplemented with human endothelial cells (HUVEC; 10%). This composition was based on histopathological observations of human RCC tissues. Cell metabolic activity was determined after 72 hours of treatment and used as a read-out for drug effects, as we noticed that spheroid size was not generally representative of drug activity due to morphological changes [30]. 3D cultures were highly sensitive to the ODC, although with the notable exception of ACHN and individual drugs also appeared more active in 3D cultures (Figure 1F,G, Figure S1.3.1–2). The observation that the activity of erlotinib, in particular, is much more potent in 3D conditions as compared to 2D was confirmed in a mouse xenograft study of Caki-1 (Figure S1.3.3). Intercellular crosstalk is responsible for this effect [30]. Furthermore, reducing the doses by 50% did not impact overall activity (Figure S1.3.2–3), in line with our previous observations [31]. This further underscores the strength of the s-FSC procedure, which searches for an optimal combination where activity is conserved over a wider range of combinations. 

### 2.2. Optimized Drug Combinations Trigger Cell Cycle Profile Changes Mediated by RPS6

Effector mechanisms of drug activity were initially assessed by cell cycle analysis profiling. In general, treatment with erlotinib or dasatinib had little effect on cell death induction. However, when axitinib was present in the ODC, it appeared to be the main driver of cell death induction by the ODC (Figure S2.1).

**Figure 2:**
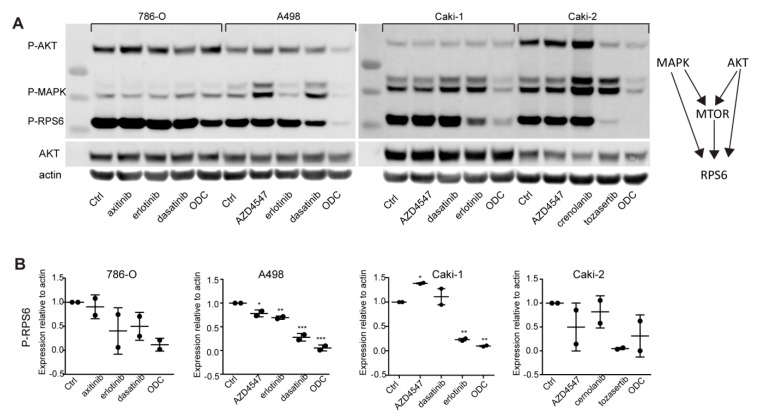
ODC induce cell cycle inhibition mediated through RPS6. Western blot analysis (**A**) and densitometric quantification (**B**) of monotherapy and ODC treated RCC, covering major cellular survival pathways that converge to RPS6. Clear inhibition of RPS6 phosphorylation is seen in all cell lines. Experiments were performed in duplicate and representative blots are shown. *: *p* < 0.05, **: *p* < 0.025, ***: *p* < 0.01.

In the next step, we investigated the activity of the three major cell proliferation/survival pathways, namely PI3K/AKT, MAPK and mTOR after the treatment with ODC and corresponding IC_20_ monotherapies. In general, ODC but not individual drugs majorly inhibited the phosphorylation of MAPK (ERK1/2) and RPS6, as shown by western blotting (Figure 2, Figure S2.2). Not unexpectedly, some monotherapies seem to increase pMAPK and or pAKT expression in selected cell lines, which may be the result of a rebound effect, while the ODC counteracts such a response. Although RPS6 is known to be directly downstream of mTOR signaling, which was not targeted by any of the drugs in the RCC lines, it can be affected by upstream AKT and MAPK signaling [32].

Taken together, these data show that although the single drugs exert only minimal effects on activity of the major cellular survival or proliferation pathways, the ODC is highly effective in suppressing these processes. Although different combinations are used, the selection for phenotype culminates in similar suppression of key effector mechanisms relating to RPS6, which controls cell growth and proliferation [33].

### 2.3. Phosphoproteomic Profiling Reveals the Molecular Signature of Active Drug Targets in RCC Lines

**Figure 3:**
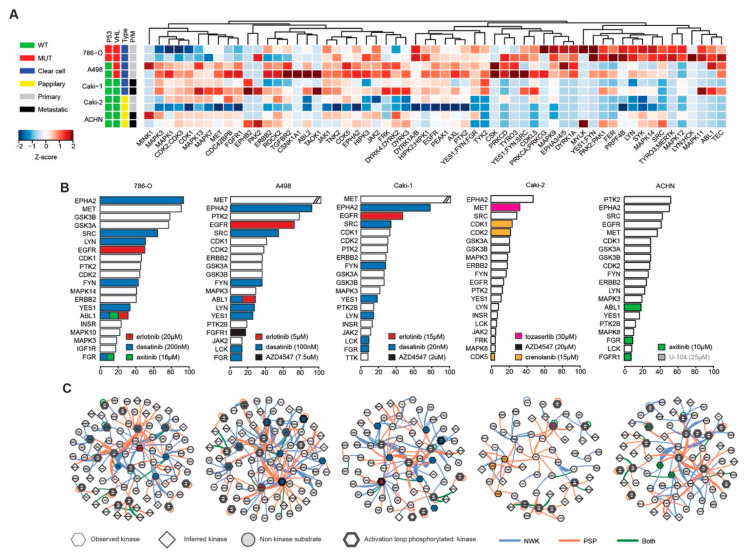
Phosphoproteomic profiling of RCC lines. (**A**) Heatmap of active (phosphorylated) protein kinases expressed in RCC under routine culture conditions. Samples are color coded according to column Z-scores. (**B**) Integrated inferred kinase activity analysis (INKA) ranking of kinase activity based on both kinase and substrate data for all RCC. Kinases are color coded for targeting drugs in the respective ODCs. (**C**) Network analysis of proteins with a positive INKA score. Drug targets are color-coded as in (**B**), and shaped according to INKA evidence. Blue lines indicate evidence by NWK, orange by PSP and green by both. A representative example of the duplicate analysis is shown for (**B**,**C**).

In order to further unravel the signaling pathways underlying effects of the ODC, the phosphoproteomes of all cell lines were interrogated using mass spectrometry-based analysis of enriched phosphopeptides. Following sequence data base searching, phosphopeptide data were aggregated to represent individual phosphorylated proteins, and phosphorylated kinases (phosphokinases) were subsequently identified. Unsupervised clustering analysis of phosphokinase expression (based on spectral counts) was performed to explore drug effects (Figure 3A). This analysis clearly showed that phosphokinase expression in the different cell lines diverges. Since kinase phosphorylation alone is only a partial measure of kinase activity, we included data on kinase activity using the novel metric of Integrative Inferred Kinase Activity (INKA) analysis [26]. This shows that despite the apparent heterogeneity in kinase phosphorylation, INKA analysis reveals remarkably similar profiles of active kinases in the different RCC cell lines. In all RCC cell lines, MET, EPHA2, PTK2 and SRC are among the top ranked active kinases (Figure 3B, Figure S3.1.1, Figure S3.1.2), while in EC-RF24 cells PTK2 and EPHA2 show a high ranking (Figure S3.1.3. In Figure 3B, the top ranking kinases are color coded for drug-target activity (www.proteomicsdb.org), which shows that the identified ODC generally target the most active kinases, such as EPHA2 and SRC by dasatinib, EGFR by erlotinib and CDK1 and -2 by crenolanib [34]. However, not all drugs used in our screen are targeting kinases, such as U-104 in the ODC of ACHN which targets CA9. Network analysis of active kinases and their substrates shows that the top INKA kinases are highly connected and present as central hubs (e.g., SRC), comparable in all cell lines (Figure 3C, Figure S3.2.1–6). 

### 2.4. Activated Kinase Profile Similarity in RCC

**Figure 4:**
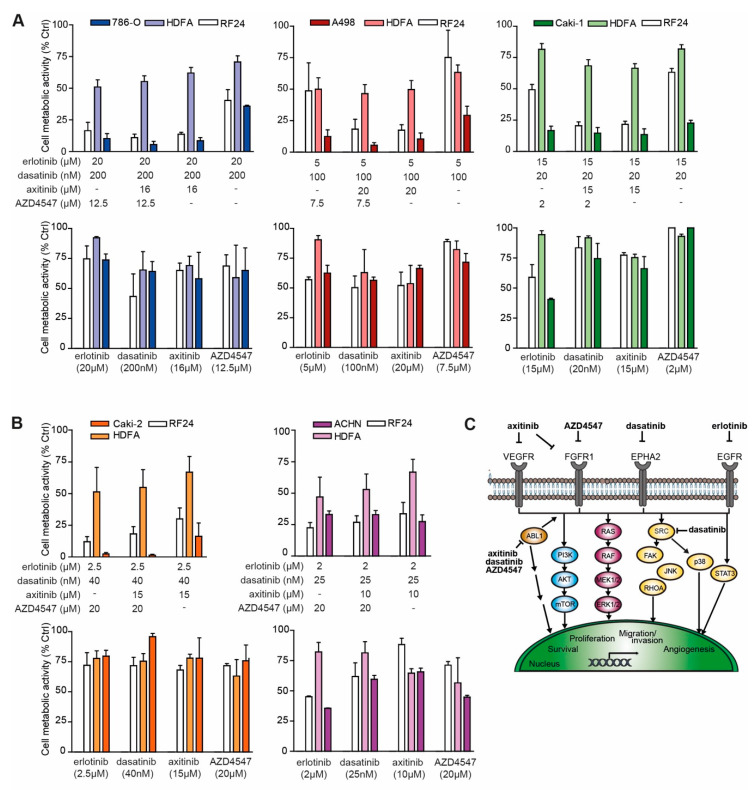
A common drug combination active in RCC cell lines. (**A**) Erlotinib and dasatinib, alone or combined with AZD4547 and/or axitinib were tested in the clear cell RCC lines that all selected for an ODC containing these compounds. Concentrations used per cell line were as used in the s-FSC. The same combinations were applied to activated endothelial cells (EC-RF24) and normal human dermal fibroblasts (HDFA). In the bottom panels, the monotherapies are displayed, which show only minimal inhibition. Experiments (N = 3–4) were performed in triplicate and mean ± SEM are presented. (**B**) Erlotinib and dasatinib, combined with AZD4547 and/or axitinib as 3- and 4-drug combinations tested in pRCC. Caki-2 and ACHN show notable sensitivity to these combinations while their original ODC selected (partially) different drugs. Experiments (N = 3–4) were performed in triplicate and mean ± SEM are presented. (**C**) Schematic of the major cellular pathways and TKI interactions for the common RCC drug combination.

The observation that the kinase activation profiles are rather similar between the ccRCC and pRCC cell lines, suggests that common drug interaction mechanisms may be effective in all RCC, and that a common RCC drug combination could be devised. We therefore tested the combination of erlotinib, dasatinib and AZD4547 or axitinib, constituting the ODC in ccRCC (Figure S4.1.1–2). To account for effects on the tumor vasculature, EC-RF24 was tested in parallel with the same drug combinations, and the fibroblast cell line HDFA was analyzed as representative of non-malignant tissue. Exchanging AZD4547 for axitinib and v.v. in ccRCC resulted in comparable growth (2D) inhibition, but inclusion of axitinib resulted in stronger reduction of EC-RF24 cell viability. HDFA are only moderately affected by the different combinations, and the AZD4547 vs. axitinib combinations had no differential effects on fibroblasts, indicating the presence of an adequate therapeutic window (Figure 4A). In addition, combining only erlotinib and dasatinib was much less effective. The pRCC cell lines used in this study converged in the s-FSC to a different set of drugs in the ODC as compared to the ccRCC, but proved particularly sensitive to both common RCC drug combinations (Figure 4B). Interestingly, using these combinations in ACHN, essentially exchanging U-104 with dasatinib and erlotinib, considerably improved effectivity in this rather resistant cell line. Inspection of s-FSC iteration data revealed that combinations of these drugs were already active in these cell lines during the screen phases, but were later excluded (Table S2. Of note, erlotinib and AZD4547 showed synergistic activity in both Caki-2 and ACHN (Figure S1.2.4–5), and the drugs presented as part of the common RCC drug combination were among the most frequently selected drugs in each cell line following the two rounds of the s-FSC, resulting in the best performing set of 4 drugs (Figure 1A–D; Figure S1.2.1–6). In terms of efficacy, each of the two common RCC drug combinations presented similar efficacy in all cell lines, while monotherapies showed minimal effects, and could thus represent an effective broad treatment approach for various RCC types (Figure 4C). 

### 2.5. Kinase Inhibition Effects from Combination Therapy Revealed by Phosphoproteomic Analysis 

**Figure 5:**
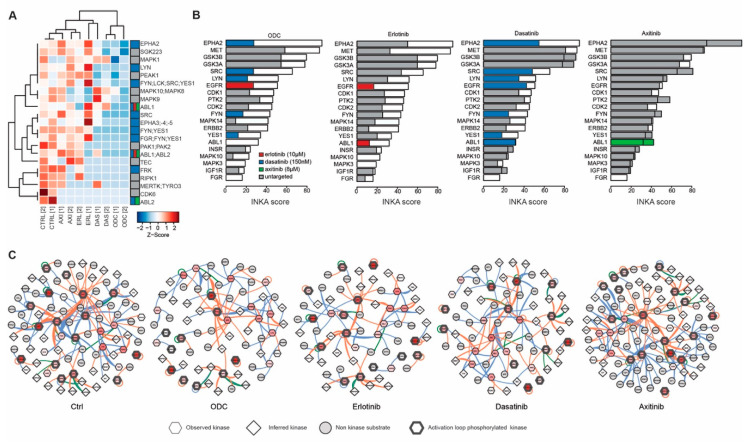
Effective protein kinase inhibition by ODC revealed by phosphoproteomics. (**A**) Heatmap of phosphokinase expression in 786-O treated with monotherapies and ODC which showed a significant difference between ODC and control. Note that the inhibitory effect of the broad spectrum inhibitor dasatinib is dominant in the ODC given their close clustering. (**B**) INKA profiles of untreated 786-O (white bars; outlined) with profiles after the indicated treatments superimposed in color. Kinases are color coded by the respective drugs. Absence of staining implicates the kinase was not present in the top 20 INKA scores after treatment, and untargeted kinases are shown in grey. (**C**) Network plots of measured kinases, substrates and inferred kinases in INKA analysis before and after treatment. Blue lines indicate evidence by NWK, orange by PSP and green by both. Drug targeted kinases are color coded in red by increasing INKA score. Representative plots of duplicate analyses are shown in (**B**,**C**).

In order to gain insight in the kinase activity before and after ODC treatment in comparison to monotherapies, 786-O cells, which are highly representative of clinical RCC, were subject to phosphoproteomics profiling and INKA analysis. Differential expression of phosphorylated kinases was tested between ODC and control treated cells (Figure S5.1.1–2). Phosphokinase expression levels under all treatment conditions are clustered in Figure 5A. The sample sub-clustering of ODC treated samples with the dasatinib treated samples clearly points to a dominant role for dasatinib in this ODC, whereas the effects of erlotinib and axitinib as monotherapies are generally mild as can be seen from the more proximal clustering along with the control treated samples (Figure 5A, Figure S5.1.1–2). The impact of the monotherapies and ODC on phosphokinase activity rather than phosphokinase expression were visualized in ranked INKA plots representative of untreated 786-O cells, overlayed in color with the INKA score after treatment (Figure 5B, Figure S5.2). ODC treatment results in pronounced inhibition of the targeted kinases, as indicated by the dominant white space in the bars. Treatment with dasatinib predominantly affect the activity of its reported targets, whereas monotherapy with erlotinib has a more widespread effect on kinase activity. In contrast, monotherapy with axitinib had little effect on the most active kinases.

Network analysis taking both kinases and substrates into account (Figure 5C, Figure S5.3.1–5) paints the picture of a dense interrelationship of kinases and their substrates in 786-O cells, which is considerably reduced with ODC treatment. It also signifies that following the ODC treatment, a number of kinases remain active and connected, which could contribute to therapy resistance. Indeed, phosphokinase expression following ODC treatment (Figure S5.1) reveals that different proliferation mediators (CDK1, -2,-3,5; DYRK1A, -2, -4), as well as MET, PTK2 (FAK), JAK2 and AXL are increased, of which several have been linked to therapy resistance towards different TKIs [35,36,37]. 

### 2.6. Addition of MET Inhibitor Improves the Activity of ODC

**Figure 6:**
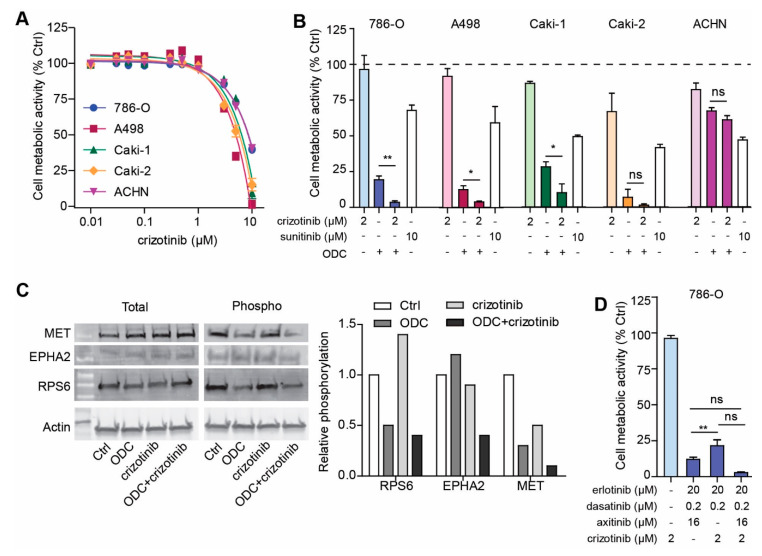
Targeting remaining active kinases further enhances ODC activity. (**A**) Dose response curves of the MET and PTK2 targeting TKI crizotinib. All RCC lines show similar sensitivity to monotherapy of this compound. Experiments (N = 1) were performed in triplicate, and fitted curves are shown. (**B**) 2 µM crizotinib was selected and displayed 5–30% inhibitory activity as single drug. When added to the original ODC, significant further inhibition of cell metabolic activity is observed in 786-O, A498 and Caki-1. Experiments (N = 3) were performed in triplicate, and mean ± SEM are shown. (**C**) Western blot of phosphoproteins after treatment with ODC, crizotinib or the combination thereof. (**D**) Exchange of axitinib for crizotinib does not improve over the original ODC in 2D cultures in 786-O. Experiments (N = 3) were performed in triplicate, and mean ± SEM are shown. ns: not significant, *: *p* ≤ 0.05, **: *p* ≤ 0.01.

Phosphoproteomics analysis of 786-O shows that PTK2 and MET remain relatively active kinases after ODC treatment, as the most active kinases (EphA2, SRC and EGFR) are effectively inhibited by dasatinib and erlotinib (Figure 5B, Figure S5.2). Therefore, we hypothesized that we could further enhance the ODC effect by including an inhibitor targeting PTK2 and MET. Crizotinib was selected as it efficiently targets MET as well as PTK2 (Figure S6.1). Dose response analysis revealed that all RCC lines are comparably sensitive to crizotinib (Figure 6A). We chose the dose of 2 µM as this represented a concentration with a similar activity (approximately IC_20_) as used with the other drugs in the s-FSC. We tested the ODC with and without crizotinib, and show that addition significantly increases ODC activity in 3/5 cell lines. For Caki-2 and ACHN, the addition of crizotinib slightly, but non-significantly potentiates their original ODC activity (Figure 6B). Furthermore, the drug combinations clearly outperform the use of sunitinib (10 µM) as monotherapy (Figure 6B). In 3D spheroids, addition of crizotinib also tended to enhance the ODC effect (Figure S6.2), albeit non-significantly as the ODC were already much more potent in 3D as compared to 2D assays in vitro.

To confirm mechanism of action of the ODC and ODC + crizotinib, western blots with cell lysates from treated 786-O cells were probed with anti-phospho specific antibodies. Figure 6C shows clear suppression of phosphorylation of the main targeted active kinases EPHA2, MET and RPS6 in the ODC where crizotinib was added as compared to the original ODC. PTK2 could not be detected. 

Interestingly, merely exchanging axitinib for crizotinib (to the combination of erlotinib, dasatinib and crizotinib) was actually less effective than the original ODC in 786-O (Figure 6D). These data suggest that while analysis of phosphokinase activity may provide clues for effective combination treatment, this may not fully cover the more complex intrinsic cellular phenotypes which are the key determinant of drug combination efficacy. 

## 3. Discussion

In current clinical cancer management, it is increasingly recognized that combining treatment regimens will lead to improved efficacy and may combat the occurrence of therapy resistance. Many studies have investigated various methods to optimize drug combinations for cancer treatment, which have the major drawback of requiring extensive experimental data generation or computational power [38,39]. We have previously presented the streamlined-feedback system control (s-FSC) methodology [21,22], which uses statistical design of experiment and stepwise linear regression to arrive at an optimized drug combination (ODC), based on phenotypic outcome. Major advantages of this method are the independence of known drug action mechanisms, as well as the minimal experimental input required when compared to systematic drug and drug combination testing in any given bioassay. In this work, we used s-FSC to investigate the effective drugable landscape of renal cell carcinoma (RCC). 

In this study, we used five divergent and commonly used RCC cell lines that represent human RCC phenotypes [27], in an s-FSC-based search to find ODC based on a combination of two to three drugs, dosed up to a maximum of the cell-line specific IC_20_. Moreover, since angiogenesis is an important hallmark of RCC, and since different agents included in the initial drug set targeting VEGF/VEGFR axis are in clinical use for RCC [40], we included the angiogenic human cell line EC-RF24 [28] in this screen as well. It was anticipated that this cell line based search could represent a precision medicine approach to identify patient tailored ODC. Indeed, all cell lines were shown to be sensitive to different ODC. However, commonalities between RCC cell lines in general, but also among clear cell RCC vs. papillary RCC, were observed.

Clear cell RCC and papillary RCC are generally characterized by loss of chromosome 3p (ccRCC) and gains of chromosomes 7 and 17 (pRCC) [41]. ccRCC is furthermore predominantly associated with high VEGF expression [41], and indeed, in all ccRCC cell lines VEGF/VEGFR axis inhibitors were selected for in the ODC. The selection of U-104, a CA9 inhibitor in the pRCC cell line ACHN can be explained by its location on chromosome 17q, as amplification can lead to overactivation of this enzyme. Similarly, tozasertib, targeting a.o. AURKB localized on chromosome 17, was selected for in Caki-2. As aurora kinases are serine threonine kinases, and carbonic anhydrases are metabolizing enzymes, these are not present in our tyrosine based phosphoproteomics analysis, precluding a conclusive statement regarding the relationship between drug sensitivity and protein activity.

Our data show that all RCC cell lines were sensitive to a combination of drugs (Figure 1), which was in general mediated by a convergence in signaling towards the cell growth and proliferation mediator RPS6 (Figure 2). Following phosphoproteomics analysis to further delineate the underlying molecular events that mediate the effectiveness of the ODC, we observed that all RCC cell lines shared 75% of their top 20 most active kinases (Figure 3 and Figure S3). In addition, the s-FSC arrived in ccRCC cell lines unequivocally at ODC containing erlotinib, dasatinib and either axitinib or AZD4547 (Figure 1, Figure S1). As these combinations target the most active kinases, and hence multiple pathways in all RCC cell lines (Figure 3), we reasoned that the combination of these compounds could be active in all RCC cell lines regardless of their genetic background and phenotype. Indeed, these combinations were unanimously effective. Even in ACHN, which appeared to be a notoriously therapy-resistant cell line and which was previously described to be insensitive to dasatinib [42]. Previously, we demonstrated that the combination of erlotinib, dasatinib and axitinib was highly synergistic, even after important dose reductions [31]. Fibroblasts were relatively insensitive for these combinations, while endothelial cells overall proved to be more sensitive to combinations containing axitinib as compared to those containing AZD4547 (Figure 4). When comparing the target spectrum of these latter two compounds (Figure S4.1.1–2), it can be noted that axitinib targets a broader spectrum of kinases than AZD4547, and in particular several important for cell survival (AURKA and -B, PLK4), and shows more overlap with erlotinib targeted kinases, which may enhance its effect. 

To gain more insight in the underlying actions of the ODC, changes in phosphokinase expression as well as activity were addressed. This showed that the ODC clearly suppresses a large part of the kinase-substrate interactions that are dominant in 786-O. The added value of INKA analysis becomes clear when looking at phosphorylation of EGFR which hardly differed between treatment conditions, while its INKA score was reduced by >50% (Figure 5, Figure S5.2). This indicates that the mere phosphokinase expression levels underestimate the more inclusive effects of drug targeting in a physiologic system. Since MET and PTK2 activation both remained high after ODC treatment of 786-O, we reasoned that by targeting these kinases with an additional TKI, we could further enhance our combination effect. MET and PTK2 inhibitor crizotinib was therefore selected and added to the ODC. The latter showed improved efficacy over ODC in most of the cell lines, accompanied by inhibition of MET phosphorylation (Figure 6, Figure S6.1). Our data are in line with a recent study by Haake et al., who performed phosphoproteomics analysis on RCC samples as well as RCC cell lines. The most abundantly expressed phosphoproteins generally showed a high INKA score in our samples, including SRC, ABL1/2, PTK2 and MET [35]. Indeed, MET activation has been recognized in RCC and linked to resistance to VEGF(R) inhibition [35,43], and combination treatment with axitinib and crizotinib was highly effective in different models of RCC [43]. Interestingly, cabozantinib, which is a TKI directed at VEGFR2, MET and AXL (the latter being activated by anti-angiogenic TKI treatment [37]), shows superior clinical efficacy in both treatment naïve as well as VEGF inhibition refractory RCC [20], further underscoring the importance of combinatorial targeting. 

Proper drug selection and dosing is not trivial. A priori information of phosphokinase activity is highly valuable to guide drug selection for use in s-FSC. Many selectively designed TKIs show off-target effects, though these might actually contribute to drug efficacy as we recently showed for crenolanib [34]. In Figure S8, the top 20 most active kinases in 786-O are presented along with all reported drugs in proteomics DB that target these kinases. Different selection procedures for drugs targeting these kinases could be followed. Either highly selective drugs, targeting specific kinases in one or multiple pathways could be included in a screen, or drugs that target multiple active kinases simultaneously could be selected. Very recently Cheng et al. [44] presented the concept of designing combination therapies based on network interactions between drugs and proteins. Only drugs that overlap separate areas (complementary exposure) of the so-called diseasome (i.e., a network of protein interactions that define disease) showed consistent effect in historical analysis of FDA approved antihypertensive drugs. Our data are in line with these observations as our s-FSC-selected ODC show very little overlap in kinase targets. Major challenges however remain as to how exactly define a diseasome [44,45,46,47]. Here we coupled pTyr-based phosphoproteomics to INKA analysis as most drugs chosen for the phenotypic screen were affecting tyrosine kinases. Yet, tyrosine phosphorylated proteins constitute only 1% of the phosphoproteome, so only a partial picture is formed of the disease network of RCC based on these data alone. Global phosphoproteomics capturing pSer and pThr events as well as gene and protein expression levels, (epi)genetic events and/or metabolic changes could undoubtedly be incorporated as well for a more complete molecular understanding. Multi-omics integration of such data into a comprehensive cancer network could be useful for selection of drug combinations by pointing to relevant targetable hubs [44,45,46,47]. However, we observed different drug interactions in our panel of cell lines despite similarities in molecular profiles, suggesting that phenotype driven selection of drug interactions and synergistic effects with s-FSC takes into account cellular mechanisms that may not be obvious from static, i.e., baseline (untreated), cellular profiles alone. As such, perceived non-optimal candidate drugs, including non-TKIs, could prove to be effective in combination approaches, through non-expected mechanisms of action, as we have demonstrated before [48]. 

Our data show that most drugs were more effective in 3D spheroid cultures than in 2D monolayer cultures, which is remarkable since the supporting fibroblasts and endothelial cells in the heterotypic spheroids were only moderately sensitive to the drugs and ODCs. This suggests that the tissue architecture plays an important role in drug efficacy. Indeed, we observed that erlotinib showed much more potent activity in vivo than in vitro, precluding us from efficiently testing the ODC in a mouse model. Dose reductions, while retaining similar combinations, did not appear to reduce activity, stressing the robustness of the ODC selected by s-FSC based on phenotype [31,49]. Thus, with regard to the application of drug combinations in clinical practice, combination therapy can be initiated at low doses with minimal risk of losing synergistic activity. 

To further increase translatability of our findings, patient-derived material is subject of ongoing studies [50] to test the feasibility of selecting ODC using s-FSC for tailored treatment. Such studies can also address the interplay with immune infiltrate. RCC is immunogenic and responsive to immunotherapy, while at the same time the tumor microenvironment is highly immunosuppressive, partly as a consequence of active angiogenesis. Anti-angiogenic treatment strategies are in fact immunomodulation, and can synergize with immunotherapy [19,51,52,53,54]. While recent studies demonstrated superiority of immune checkpoint inhibitor combination therapy over single agent tyrosine kinase inhibitors in clinical management of RCC [19], additional room for improvement clearly persists. Notably, combination therapies could prevent or overcome resistance against single agents, allow for relative dose reductions and contribute to better management of adverse effects. Assessment of the interplay between intercellular synergistic effects and intracellular synergistic TKI activities in more complex in vitro models that recapitulate human RCC such as organoids, will further elucidate optimal translatable treatment combinations.

Although s-FSC does not require prior knowledge of drug effector mechanisms, molecular profiling to uncover dominant pathways in cells or tissues is helpful in refining initial drug selections. Taken together, phenotype based screening for optimized drug combinations proves highly effective in defining drug combinations for different cell types. 

## 4. Materials and Methods 

### 4.1. Phenotype Screen for Optimized Drug Combinations (ODCs) by s-FSC

Details on compounds and cellular assays to evaluate drug effect, as well as details on s-FSC data modeling are presented in the Supplementary Information. 786-O, Caki-1, Caki-2, ACHN and A498 (human RCC) (ATTC, Manassas, Virginia, USA), were included. A standardized cell metabolic assay was used for the evaluation of cell line specific dose–response curves and estimations of IC_20_ (Figure S1.2, Table S1.1), as well as for the evaluation of combinations [21].

### 4.2. Evaluation of ODCs

ODCs were subsequently evaluated in additional assays, full details are described in the Supplementary Information. Heterotypic 3D co-cultures were obtained by mixing the RCC cells with HDFA in a ratio of 3:1 (4500 and 1500 cells, respectively), followed by addition of 10% HUVEC (600 cells) per spheroid. Cell cycle distribution was assessed based on flow cytometry analysis of cellular DNA content as previously described [55]. 

### 4.3. Animal Procedures

All animal procedures were performed and approved by the Institutional Ethical Committee of Animal Care in Geneva and the Swiss Cantonal Veterinary Office (Authorization number GE-2-17). All the procedures were carried out using appropriate guidelines and regulations on the animals for welfare of the animals. See Supplementary Methods for details.

### 4.4. Phosphoproteomics and INKA Analysis

Cells were cultured to near-confluence and left untreated or were exposed (786-O) to drugs as indicated in Figure 5 for 2 h. The full workflow is detailed in the Supplementary Methods section, and was essentially performed as described before [26]. Briefly, proteins were digested with trypsin, phosphopeptides were pTyr immunoprecipitated and subject to LC-MS/MS, followed by searching MS/MS spectra against Swissprot human proteome using MaxQuant 1.6.4.0. Normalized spectral count data for all kinases are presented in Table S3.1. INKA was performed as recently described [26], and INKA scores are presented in Table S3.2. 

For interpretation and visualization of phosphokinase expression, normalized count data were used. Protein-protein interactions were analysed using String (www.string-db.org), and visualized with Cytoscape (v3.7.1). Enrichment analysis was done using Enrichr (http://amp.pharm.mssm.edu/Enrichr) [56]. Drug-target interactions were analysed and visualized using data previously generated by Klaeger et al., based on cell-free assays [57] (www.proteomicsdb.org). For ccRCC cell lines, effective inhibitory percentages per kinase are shown in Table S3.3, using the applied drug doses per cell line.

### 4.5. Statistical Analysis

Data are presented as means of multiple independent experiments. Error bars represent standard error unless specified otherwise. Statistical significance was determined in GraphPad Prism^®^ (version 5.01, www.graphpad.com/scientific-software/prism/) using either one-way ANOVA with either Dunnett’s post hoc multiple comparison test (when comparing to a single control condition) or with Bonferroni’s post hoc multiple comparison test (when comparing multiple selected conditions), or using student’s *t*-test, with * *p* < 0.05 and ** *p* < 0.01.

## 5. Conclusions

In this study we have shown that combining s-FSC with a phosphoproteomic profiling approach provides valuable insight in targetable kinase activity and allows for the identification of superior drug combinations for the treatment of RCC. Next to the identification of unique drug combinations for each cell line, we identified a common RCC drug combination applicable to multiple cell lines, guided by kinase activity profiles of the cells. Finally, we uncovered compensatory mechanisms following drug treatment that could be readily targeted by additional drugs. Ideally, similar analyses should be performed using freshly resected RCC patient material to demonstrate the superior value of combined targeted therapy in the clinical management of RCC patients.

## 6. Patents

The authors (P.N.-S. and A.W.) are the inventors on pending patent (EP19199136) and issued patent (P.N.-S., A.W., and A.W.G. WO2015136061A3) on methods of drug combination therapy. Other authors have no conflict to disclose.

## Supplementary Materials

The following are available online at https://www.mdpi.com/2072-6694/12/9/2697/s1, Supplementary Methods, Figure S1.1.1: Drug selection, Figure S1.1.2: Monotherapy dose response curves, Figure S1.2.1–6 Screening for optimized drug combinations, Figure S1.3.1: Spheroid morphology after drug treatment, Figure S1.3.2: Dose sensitivity in 3D mono- and coculture systems, Figure S1.3.3: Monotherapies and combinations of drugs in Caki-1 xenografts in mice, Figure S2.1: Effects of ODC on cell cycle profile, Figure S2.2: Mechanism of action of drug combinations, Figure S3.1.1: Duplicate INKA analysis of RCC lines, Figure S3.1.2: Overlap of top 20 INKA kinases in 5 RCC lines, Figure S3.1.3: Phosphoproteomics analysis of EC-RF24, Figure S3.2.1–6: INKA network plots of RCC lines and EC-RF24, Figure S4.1.1–2: Protein drug interaction maps, Figure S5.1.1: Phosphokinases (pTyr) downregulated after ODC, Figure S5.1.2: Phosphokinases (pTyr) upregulated after ODC, Figure S5.2: INKA profiles after monotherapies and ODC in 786-O, Figure S5.3.1–5: INKA network plots of monotherapies and ODC in 786-O, Figure S6.1: Protein-drug interaction maps, Figure S6.2: Limited additional effect of crizotinib addition in 3D cultures, Figure S7.1: ODC in EC-RF24, Figure S8: Heatmap of EC_50_ of drugs inhibiting most active 786-O kinases, Figure S9.1–3: Original Western blot images and reference to Figure 2 and Figure S7.1, Figure S10.1–2: Original Western blot images and reference to main figure 6, Table S1.1: IC20 drug doses used per cell line in s-FSC, Table S1.2: Characteristics of the cell lines used, Table S2: Cell viability in s-FSC iteration rounds for the common RCC drug combinations, Table S3.1: Normalized spectral counts of phosphokinases, Table S3.2: INKA scores of phosphokinases, Table S3.3: Effective inhibitory % of ODC for expressed kinases per cell line in ccRCC.
